# Operando Observation of Oxygenated Intermediates during CO Hydrogenation on Rh Single Crystals

**DOI:** 10.1021/jacs.2c00300

**Published:** 2022-04-08

**Authors:** David Degerman, Mikhail Shipilin, Patrick Lömker, Christopher M. Goodwin, Sabrina M. Gericke, Uta Hejral, Jörgen Gladh, Hsin-Yi Wang, Christoph Schlueter, Anders Nilsson, Peter Amann

**Affiliations:** † Department of Physics, AlbaNova University Center, Stockholm University, Roslagstullsbacken 21, Stockholm 114 21, Sweden; ‡ Department of Physics Combustion Physics, Lund University, Professorsgatan 1, Lund 223 63, Sweden; § Division of Synchrotron Radiation Research, Lund University, Professorsgatan 1, Lund 223 63, Sweden; ⊥ Photon Science, Deutches Elektronen Synchrotron DESY, Notkestraße 85, Hamburg 226 07, Germany

## Abstract

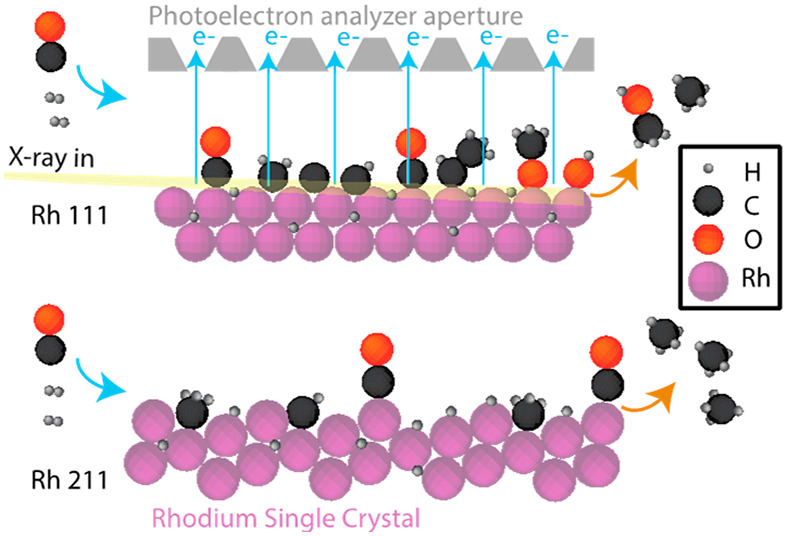

The CO hydrogenation reaction over the Rh(111) and (211) surfaces has been investigated operando by X-ray photoelectron spectroscopy at a pressure of 150 mbar. Observations of the resting state of the catalyst give mechanistic insight into the selectivity of Rh for generating ethanol from CO hydrogenation. This study shows that the Rh(111) surface does not dissociate all CO molecules before hydrogenation of the O and C atoms, which allows methoxy and other both oxygenated and hydrogenated species to be visible in the photoelectron spectra.

The mixture of CO, CO_2_, and H_2_, known as synthesis gas or syngas, can be used to produce chemicals and fuels.^1,2^ New approaches utilizing biomass production, hydrogen from water splitting, and direct capture of atmospheric CO_2_ have opened up the possibility for sustainable manufacturing routes, lowering the dependence on fossil resources.^2−5^

These processes rely on active and selective catalysts where the selectivity toward multicarbon oxygenates (referred to as C2+ oxygenates) is desired, as they have a high volumetric and gravimetric energy density and contain functional groups.^1^ Rhodium is a catalyst that has a slightly elevated selectivity toward C2+ oxygenates in general and higher alcohols in particular in comparison to other metals.^1,6,7^ The reason for the high C2+ oxygenates selectivity could be that CO adsorb onto the Rh surface both dissociatively and nondissociatively. Hydrogen atoms will sequentially attach to the C and O species to form CH_4_ and H_2_O. Before the attachment of a final hydrogen atom, however, there is a probability that the CH_*x*_ species will bind to CO rather than H and form a CH_X_CO intermediate.^6−11^ The bond formation between unsaturated hydrocarbons and nondissociated CO is proposed to be the crucial step when creating multicarbon oxygenates.

Theoretical calculations of reaction barriers significantly contributed to the proposed oxygenates mechanism. Calculated values for the ground-state and transition-state energies for every intermediate have been combined into a microkinetic model (MKM). The predicted turnover frequencies (TOFs) have been compared to experimental TOFs with relatively good agreement.^12−14^ The coverages of adsorbates depict the catalyst’s resting state, i.e., species at the minima of the potential energy surface, and provide a more direct correlation to the reaction mechanism than the TOFs of various products. In the past, experimental difficulties have prevented this determination, because it requires a combination of (1) surface sensitivity, (2) element and compound specificity, and (3) operando conditions. X-ray photoelectron spectroscopy (XPS) fulfills the first two criteria but has been very impractical to perform operando during hydrogenation reactions because of the necessity of high pressures. XPS studies of Rh and other syngas catalysts have consequently been limited to pressures around or below 0.2 mbar.^15−17^ With recent advances in XPS instrumentation and the virtual pressure cell concept, we now have the experimental tools to examine reaction conditions at pressures in the hundreds of mbar regime.^18^

We have probed the CO and CO_2_ hydrogenation reactions over Rh(111) and Rh(211) by XPS at a pressure of 150 mbar and report herein the observation of oxygenate intermediates in line with the reaction pathway expected by DFT calculations.^14^ For fcc metallic systems such as Rh, the (111) surface comprises a flat terrace of close-packed atoms and the (211) surface has a high step density.^6,13^ Comparing the two surface orientations assists in elucidating the effect of undercoordinated step edges, which are known to impact the rate of dissociation for bonds such as the C=O bond of a CO_2_ molecule.^10,19,20^ This comparison is important because the feasibility of CO and CO_2_ bond breaking on Rh(111) has been debated.^21−25^

Figure 1 shows X-ray photoelectron (XP) spectra of the C 1s region during CO hydrogenation on Rh(111) and Rh(211). The spectrum intensity, which is proportional to the surface coverage, was estimated for all spectra. The total carbon coverage on Rh(111) decreases with temperature from 96% of a monolayer (ML) at 175 °C down to 12% of an ML at 325 °C. Similarly, on Rh(211), the coverage ranges from 80.5% of an ML down to 11% in the same temperature span. The recoil of the high-energy photoelectrons and coadsorption effects may cause the peak positions to be slightly different from more conventional soft X-ray experiments.^26−29^ For a detailed technical discussion on the spectra, we refer to the Supporting Information.

**Figure 1:**
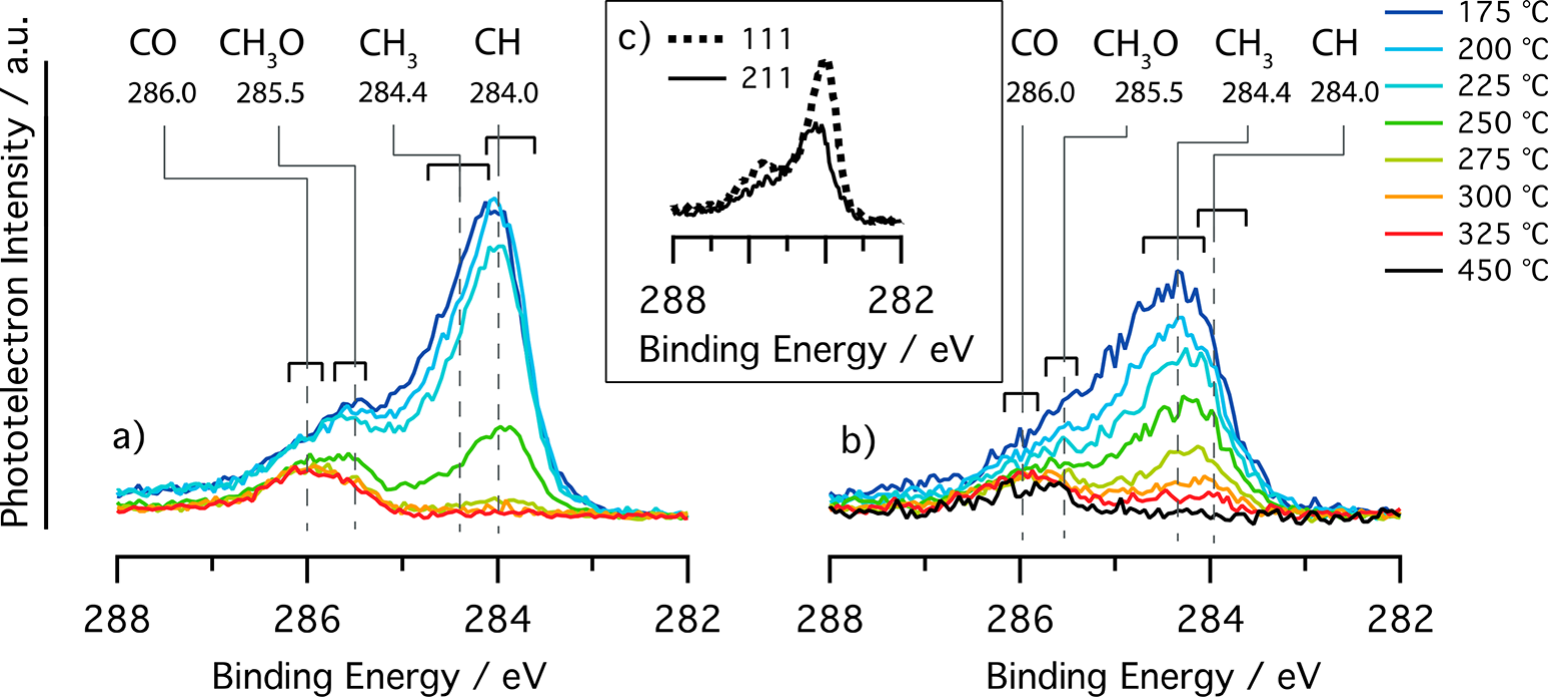
XP spectra acquired during CO hydrogenation of the C 1s region for two different Rh surfaces: (a) the (111) surface and (b) the (211) surface; (c) comparison of the 200 °C spectra for both surfaces. The CO:H_2_ ratio was 1:2, the photon energy was 4.6 keV, and the pressure was 150 mbar. A full description of the experimental parameters is available in the Supporting Information.

Four components are identified in Figure 1. The first two are the global peak maxima at the lowest temperatures, 175–200 °C, which on the (111) surface is found at 284.0 eV binding energy (BE), and on the (211) surface, it is located at 284.4 eV. These peaks correspond to differently hydrogenated CH_*x*_ fragments. We attribute the 284.0 eV peak to CH and the 284.4 eV peak to CH_3_. Other species that contribute to the spectrum intensity include atomic C, CH_2_, and C–C bonded carbon.^29,30^ The coverage of the hydrocarbon species decreases with increasing temperature, which is likely an effect of faster termination of the hydrocarbon intermediates resulting in higher reaction rates.^31^

At 275–325 °C, we observe a peak at 286.0 eV for both surface orientations related to CO in the on-top position, the energetically most favorable site.^32^ The CO coverage, as seen in Figure 2, is stable at around 7–10% of an ML on Rh(111) and 6–8% of an ML on Rh(211).

**Figure 2:**
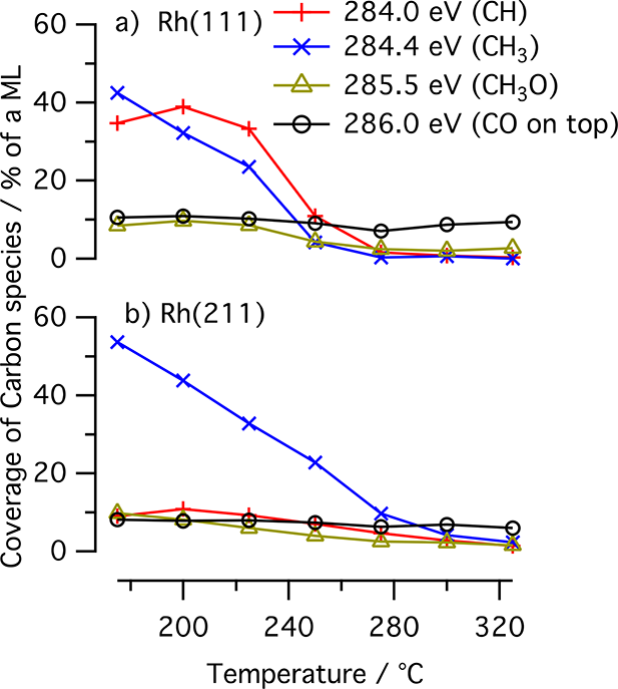
Trends in coverages of chemisorbed carbon-containing species during CO hydrogenation as obtained by fitting of the spectra in Figure 1. Results for (a) Rh(111) and (b) Rh(211). The fits are available in the Supporting Information.

Lastly, the second maximum is a peak located at 285.5 eV, and most visible at temperatures 175–250 °C on the Rh(111) surface. It is well-known that CO in hollow sites has lower binding energy than CO on-top-site. It is, however, very unlikely that those sites become populated to a large degree at coverages lower than 33% of a ML.^32,33^ Thus, we denote the peak at 285.5 eV as the methoxy (CH_3_O) intermediate. Other oxygenated products such as ethoxy (CH_3_CH_2_O), may contribute as well. The methoxy species is more prevalent on the (111) surface than on the (211) surface, consistent with the expectedly slower CO dissociation on the (111) surface. The CO and methoxy species are further confirmed by the O 1s spectra provided in the Supporting Information. Some hydrocarbon fragments in the 284–284.5 region can be related to the formation of C2+ oxygenates but we cannot firmly conclude that in the XP spectra because of overlap with their monocarbon equivalents.

In Figure 2, we have plotted how the coverages of the four fitted components change with temperature. We note a decrease of all components with temperature except for the 286.0 eV peak, which corresponds to CO. We have minor indications of hydrocarbon and methanol formation in the online mass spectrometry (MS) acquisition provided in the Supporting Information.

XP spectra of the C 1s region during CO_2_ hydrogenation are shown in Figure 3. We can discern two main peaks, one corresponding to CH_*x*_ at 284.0 eV and a second peak corresponding to oxygenated C species around 286.0 eV. Qualitatively, the same types of surface species are present in the CO_2_ hydrogenation reaction as for CO hydrogenation, but with significantly lower coverages. The total coverage of carbon species on both Rh(111) and Rh(211) are between 2 and 10% of an ML. The lower coverage for the CO_2_ hydrogenation reaction can be clearly seen in Figure 3c, where the adsorbate spectra of the two reactions are compared. The weak binding of CO_2_ to Rh results in a short residence time on the surface and, in turn, low probability for CO_2_ dissociation.^17^ The higher coverage of the (211) surface compared to the (111) surface is expected because the dissociation rates of CO_2_ have been estimated to be faster because of lower barriers on the (211) surface.^34,35^

**Figure 3:**
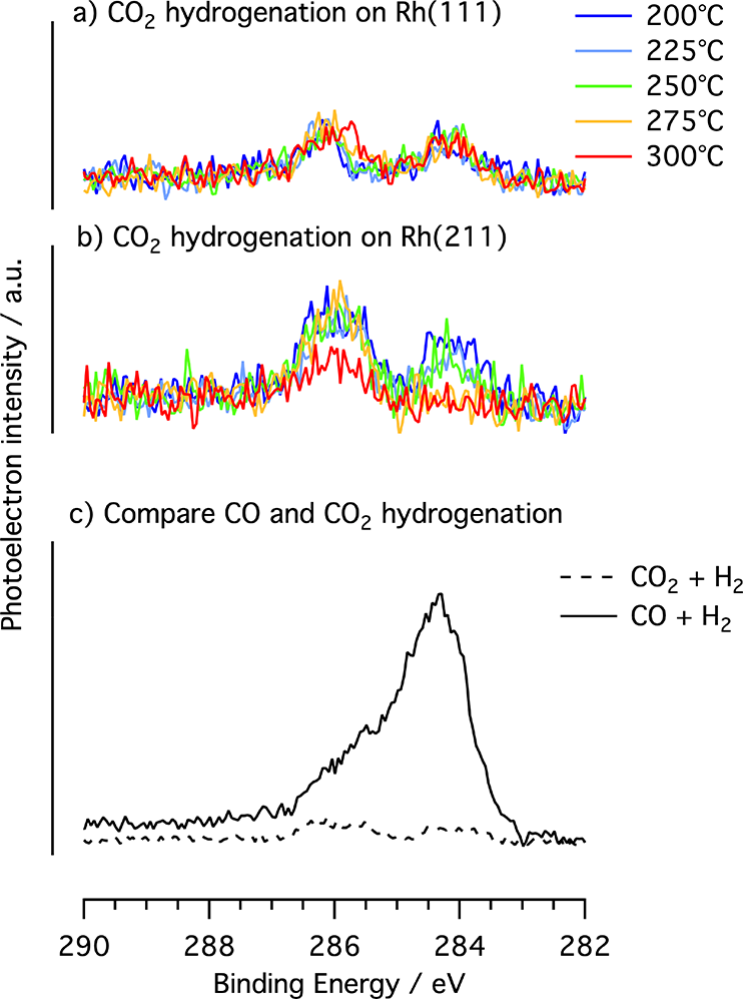
XP spectra of the C 1s region during CO_2_ hydrogenation on (a) Rh(111) and (b) Rh(211). (c) Comparison of the spectra acquired during CO and CO_2_ hydrogenation on Rh(211) at *T* = 200 °C. The pressure in all spectra is 150 mbar and the CO_2_/CO:H_2_ ratio is 1:2.

## Implications of Results

The presence of methoxy species (or other oxygenates) indicates that some CO is almost fully hydrogenated before dissociation. The fact that the coverage of CH_*x*_ species is temperature dependent tells us that other CO molecules are actively dissociated in the presence of H_2_. This holds for both the stepped (211) surface and the more terrace-like (111) surface. These observations support the hypothesis that CO is hydrogenated both before and after dissociation on Rh and is consistent with DFT calculations proposing that the hydrogen-assisted dissociation step occurs via the CH_3_O intermediate.^14^ The observation of CH_3_O may also be indicative of methanol production, consistent with the MS data in the Supporting Information.

Theoretical calculations have indicated that the Rh(111) surface likely has higher selectivity than the (211) surface toward C2+ oxygenates. The reasoning is related to the higher activity for the competing methanation reaction on the (211) surface, whereas C–C coupling reactions require a coexistence of nondissociated CO and nonsaturated CH_*x*_-species, and it has been shown that the rate for C–C coupling increase with increasing CO coverage.^11,13^ The dominance of CH_*x*_ species in Figure 1 below 250 °C on both surface orientations indicate a low-CO-coverage regime which favors monocarbon products. Still, it is interesting to examine the prerequisites for C–C coupling of the examined reaction conditions. We note a higher degree of hydrogen saturation on the (211) surface, whereas the (111) surface at lower temperatures has a higher fraction covered in low-saturated hydrocarbons. DFT studies have shown that CO insertion into CH is has lower barriers than insertion into CH_3_.^6,36^ Both CO and CH_*x*_ show in general higher coverage on the (111) surface. Additionally, a resting state of mainly CH means less immediate competition from the termination reaction producing hydrocarbons. These observations combined lead us to conclude that the prerequisites for C–C coupling are favorable on the (111) surface around 250 °C or below.

The CO coverage dependence could be one of the reasons high pressure is needed to obtain more C–C coupling in CO hydrogenation. Another factor that has been debated is the coadsorption with H_2_. Low energy electron diffraction and electron energy loss spectroscopy studies of CO on Rh(111) have shown that the coverage is unaffected by the coadsorption of H_2_.^37^ Several of the DFT studies utilize this assumption in their theoretical calculations.^6,13,39^ In stark contrast, temperature-programmed desorption studies in ultrahigh vacuum of the same system have shown that the uptake of CO on Rh(111) is heavily dependent on the presence of H_2_ and that the CO coverage saturates at ∼15% of a ML hydrogen for precovered surfaces.^40^ This work supports a picture where coadsorbed H severely limits the uptake of CO on Rh surfaces, based on the following observations: We note on Rh(111) a CO-on-top coverage of 7–10% that does not decrease significantly with increasing temperature up to 325 °C. In the case of dynamic equilibrium, we would have expected the coverage to decrease with increasing temperature as the desorption rate of CO is strongly temperature dependent. Still, we only see this effect in Figure 1b, where the CO-on-top region is slightly diminished at 450 °C (see also Figure S9 in the Supporting Information). For all other temperatures, on both the (111) and (211) surfaces, we note a relatively stable coverage and consequently conclude that the CO must be at saturation coverage. At temperatures below 250 °C, the coadsorption with other carbon species will prevent the CO species from covering the surface, which may cause the CO coverage to saturate, but even above 275 °C, where CO is the only spectroscopically visible C 1s species, we observe the 7–10% coverage. We thus propose that the spectroscopically nonvisible hydrogen competes with CO for adsorption sites and causes the CO to saturate.

In conclusion, we have utilized operando XPS during CO and CO_2_ hydrogenation reactions on Rh(211) and Rh(111) catalysts for a mechanistic investigation. The surface adsorbates comprise mainly CO, hydrocarbons and methoxy radicals. We have found indications that the CO coverage is heavily affected by the coadsorption of H_(ads)_. The degree of hydrogenation is sensitive to the surface orientation, and the coverage of hydrocarbons and methoxy is temperature dependent. For the prospect of production of C2+ oxygenates, such as ethanol, a higher partial pressure of CO is required to increase the C–C coupling rate. In the currently studied reaction conditions the Rh(111) surface around 225–250 °C has better prerequisites for C–C coupling than the other studied conditions. In the CO_2_ hydrogenation reaction, we observe CO and unsaturated hydrocarbons, but at much lower coverages. In general, we conclude that insights of the presence of different adsorbates during operando studies assists in understanding selectivity in syngas reactions.

## Supporting Information Available

Detailed description of materials and methods, O 1s and Rh 3d spectra, fitted C 1s spectra, technical discussion of spectra and peak assignment, mass spectrometry data, and discussion (PDF)

## Abbreviations

DFT: density functional theory
MKM: microkinetic model
TOF: turnover frequency
XPS: X-ray photoelectron spectroscopy
XP: X-ray photoelectron
ML: monolayer
MS: mass spectrometry
