# Supplementary material for: Operando Observation of Oxygenated Intermediates during CO Hydrogenation on Rh Single Crystals
Source: J Am Chem Soc. 2022 Apr 8;144(16):7038–42. doi: 10.1021/jacs.2c00300 (PMC9052753; doi:10.1021/jacs.2c00300)
Supplement: Supplementary file 1 — ja2c00300_si_001.pdf [file ja2c00300_si_001.pdf]

Supporting Information for *Operando* observation of oxygenated intermediates during CO hydrogenation on Rh single crystals.

AUTHOR NAMES: David Degerman*[#], Mikhail Shipilin[#], Patrick Lömker[#], Christopher M. Goodwin[#], Sabrina M. Gericke[§], Uta Hejral[&†], Jörgen Gladh[#†], Hsin-Yi Wang[#†], Christoph Schlueter[±], Anders Nilsson[#] and Peter Amann[#†]

AUTHOR ADDRESS:

[#] David Degerman, Mikhail Shipilin, Christopher M. Goodwin, Hsin-Yi Wang, Jörgen Gladh, Anders Nilsson, and Peter Amann

Stockholm University
Department of Physics
AlbaNova University Center
Roslagstullsbacken 21
114 21 Stockholm
Sweden
Email: david.degerman@fysik.su.se

[§] Sabrina M. Gericke
Department of Physics
Combustion Physics,
Lund University
Professorsgatan 1
223 63 Lund
Sweden

[&] Uta Hejral
Division of Synchrotron Radiation Research
Lund University
Professorsgatan 1
223 63 Lund
Sweden

[±] Christoph Schlueter
Photon Science
Deutsches Elektronen Synchrotron DESY
Notkestraße 85
226 07 Hamburg
Germany

[†] Present addresses are given in Author information

* Corresponding Author

KEYWORDS: Reaction mechanisms, Heterogeneous Catalysis, Operando Analysis, Photoelectron Spectroscopy, Rhodium

ABSTRACT: The CO hydrogenation reaction over the Rh(111) and (211) surfaces have been investigated operando by x-ray photoelectron spectroscopy at a pressure of 150 mbar. Observations of the resting state of the catalyst give mechanistic insight to the

selectivity of Rh for generating ethanol from CO hydrogenation. This study shows that the Rh(111) surface does not dissociate all CO molecules before hydrogenation of the O and C atoms, which allows methoxy and other both oxygenated and hydrogenated species to be visible in the photoelectron spectra.

Materials and Methods

Experimental Procedures

Experimental set-up

The experimental data was obtained from a beamtime experiment between the 24th and 28th of September 2020, at Beamline P22 at the storage ring PETRA III in Hamburg, Germany. The beamtime and proposal ID for the beamtime was ID I-20191434 EC. The high-pressure x-ray photoelectron spectroscopy (XPS) endstation POLARIS was used to acquire all the XP spectra. A full description of the system is available in reference[1] but a brief description is presented here with some parameters described. The system is a custom-made instrument developed at Stockholm University for the specific purpose of analyzing catalytic hydrogenation reactions. The design is based on the concept of a stagnation flow catalytic reactor in a rough vacuum chamber, where the pressure is locally produced in a virtual pressure cell. The system is equipped with a Scienta R4000 hemispherical analyzer attached to a HiPP-2 differential pumping lens and a multichannel plate detector imaged with a charge coupling device.

Mass spectrometer data was gathered using a Hiden HAL/3F RC 301 PIC system located at the first differential pumping stage of the analyzer pre-lens. A leak valve controls the pressure to be at the highest possible working pressure for the secondary electron multiplier detector.

The distance between the sample and the electron analyzer inlet was continuously adjusted to maintain the constant pressure and to correct for the sample's thermal expansion. This was done utilizing a PID loop operating on the pressure of the first differential pumping stage, and the average distance was 20-25 μm for all the measurements.[2]

X-ray beam properties

Unless stated otherwise, all spectra have been acquired with 4600 eV photon energy, using the Si(311) double crystal monochromator. Grazing incidence (incidence angle = 0.4°) and normal emission mode was used for spectrum acquisition. The beam focus was roughly 10 x 10 μm, and the incidence angle resulted in a beam footprint of roughly 1400 mm on the sample. The grazing incidence geometry is necessary to maintain surface sensitivity while using hard x-rays. When the angle is below the critical angle for total external reflection, the penetration depth d_z of the evanescent wave will depend on the wavelength according to Equation S1.[3]

$$d_z = \frac{\sqrt{2}\lambda}{4\pi\sqrt{\sqrt{\left(\alpha_i^2 - 2\delta\right)^2 + 4\beta^2} - \alpha_i^2 + 2\delta}} \qquad \text{Equation S1}$$

Where α_i is the incidence angle and β and δ are the real and imaginary part of the refractive indicies. The penetration depth of the reflected beam using the aforementioned beamline settings was estimated to be around 16 Å. Before attenuation, the beam intensity was on the order of 10^{13} photons, with an attenuation factor of 5 for the CO hydrogenation experiments and a factor of 12 for the CO_2 hydrogenation experiments in order to avoid beam induced effects on the spectra.

Samples and chemicals

The samples are two pure Rh single crystal surfaces, cut and polished along a (111) and (211) plane. The crystals were bought from Surface Preparation Lab, both 10 mm in diameter, hat-shaped with a 7 mm polished surface. The main difference between the two single crystals are in the step density, where the (111) surface orientation comprises a terrace surface and the (211) surface contains a large number of steps. The defect concentration has been estimated for both crystals with the verdict that < 1/64 of the surface unit cells is defect. We want to emphasize, however, that the defect concentration changes after every surface preparation, and the surface may also exhibit dynamic changes during reaction conditions. The sample was heated from the back-side by a boron nitride heater and attached to a sample holder made of stainless steel type 1.4762 – AISI446. During operando measurements, the polished sample surface faces the analyzer, and the distance between the sample surface and the analyzer inlet was 25-30 μm as measured by a monochrome camera with a microscope lens attached.

The sample was cleaned by a recipe which involved a flash desorption heating to 600°C, repeated sputtering by Ar^+ at 5 kV for 15 minutes and subsequent annealing to 950 °C for 3 minutes to ensure the desired surface. Despite careful preparation a small contaminant of Si (around 4 % of a monolayer) was detected throughout the measurements. No other contaminant was detectable in the XP spectra.

Before measurement start, the surface was chemically cleaned by O_2 and H_2 at 450°C, where oxygen removed adventitious carbon and hydrogen removed potential surface oxides created by the oxygen treatment. After this chemical cleaning the sample was cooled down in 0.16 L_N/min H_2 until the intended measurement temperature was reached.

The sample gases used for the experiment were H_2, CO, CO_2, O_2, and He. H_2, CO_2, O_2 and He were of 5N purity while the purity of CO was 99.997%. H_2, CO_2 and He were delivered from 50 L 200 bar cylinders while CO and O_2 were delivered from similar 10 liter bottles to accommodate for the safety regulations at DESY. The gas cylinders were stored in safety cabinets ~20 meters of piping away from the point of the experiment. For CO_2, O_2, He and H_2 gas purifiers from SAES (model Nr. MC45) were implemented. In order to avoid formation of nickel carbonyls by CO interacting with stainless steel, a combination of copper piping and a gas purifier for CO (PALL, GLP8SIPVMM4) was used. The gases were dosed and mixed using a custom-built mixer. During operando measurements the flow was 0.20 L_N/min CO or CO_2 and 0.4 L_N/min H_2. The gas mixing unit is not a UHV system.

Data analysis

Data analysis was performed using custom scripts for IGOR PRO 6.3 and Python 3. For adsorbate peak fitting, the specqp program and graphical user interface by Dr. Mikhail Shipilin was used.[4] The peaks were fitted to Voigt shapes. For the Rh 3*p* region a Tougaard background was utilized, where the metal and gas phase peaks were fitted as Voigt shapes, while the electron energy loss features were fitted as asymmetric Voigt shapes according to reference.[5]

Spectra normalization and background subtraction

The O 1*s* and C 1*s* spectra presented in Figures 1 and 2 of the main text were normalized by the integration time and a constant background was subtracted. Inelastic scattering from photoelectrons scattering in the gas phase contribute to the background on the higher binding energy side, and can be modelled by a Shirley background, but the distinction between inelastic background and shake-up intensities from the adsorbates is hard to achieve, which is why it was left out of the presented figures in the main text. In order to do peak fitting, however, a modelling of the background is a technical necessity. Therefore, for peak fitting purposes of the C 1*s* spectrum, a Shirley background has been modelled in the fit and subtracted, neglecting the possibility for shake-up intensity to contribute.

In the combined O 1*s* and Rh 3*p* spectra, used for subtraction of the electron energy loss feature, a Tougaard background has been modelled for the fit of the spectrum. The Tougaard background has also been used for analysis of the Rh 3*d* spectra.

Calculations of surface coverage

The total coverage estimations are based on the comparison between the peak areas of the C 1*s* adsorbate region and the Rh $3d_{5/2}$ component and for oxygen on the comparison between the area of the O 1*s* adsorbate region and the Rh $3p_{1/2}$ component. An example of the overview spectra that were used for this calculation is given in figure S1.

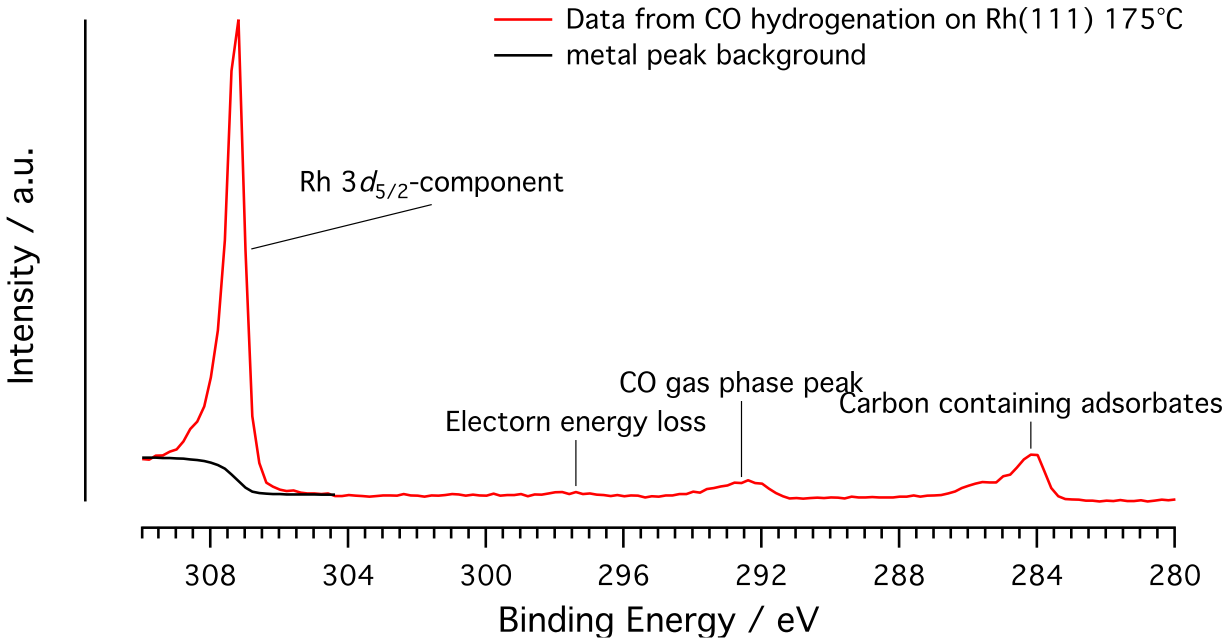

Figure S1. Overview XP spectrum utilized for the calculation of the surface coverage including the C 1*s* and Rh 3$d_{5/2}$ regions.

This method has previously been utilized for similar coverage estimations for CO oxidation reactions.[6] In order to estimate the photoelectron intensity I of a substrate of thickness t we utilize Equation S2

$$I \propto \rho\sigma I_0 \lambda' \cdot e^{-\frac{POS}{k_B T}} \cdot \int_0^t e^{-\frac{z}{d_z}} \cdot e^{-\frac{z}{\lambda}} dz \qquad \text{Equation S2}$$

In this equation ρ is the number density of the atom in the substrate and, σ is the photoionization cross section, I_0 is the intensity of the x-ray beam and λ' is the effective probing depth which depends on both the mean free path λ of the photoelectrons and the penetration depth d_z of the x-rays (see Equation S1, in the "x-ray beam properties" section). The exponential term that follows models the attenuation of the photoelectrons in the gas phase. P is the pressure, O is the inelastical scattering cross section of the gas phase and S is the travel distance of the photoelectrons in the gas phase. The integral over the thickness combines the effect of x-ray attenuation depth d_z and the photoelectron attenuation dependent on the mean free path λ within the substrate.

The surface coverage calculations performed in this work utilize a model where the atomic coverage of one adsorbate species (C or O, denoted ads in the equation) of thickness t is compared to the semi-infinite Rh substrate denoted m in the equation. This modifies the equation a bit as the treatment of two layers have to be performed separately, but simultaneously, some of the terms which are the same for both species cancel out. The result is Equation S3

$$\frac{I_{ads}}{I_m} = \frac{\rho_{ads}\sigma_{ads}\lambda'_{ads} \cdot \int_0^t e^{-\frac{z}{d_{z,ads}}} \cdot e^{-\frac{z}{\lambda_{ads}}} dz}{\rho_m \sigma_m \lambda'_m \cdot e^{-\frac{t}{d_{z,ads}}} \cdot e^{-\frac{t}{\lambda_{ads}}} \int_t^\infty e^{-\frac{z}{d_{z,m}}} \cdot e^{-\frac{z}{\lambda_m}} dz} \qquad \text{Equation S3}$$

The added exponentially decaying factors in the denominator are due to the attenuation of the Rh photoelectrons by the adsorbate layer. In order to extract the coverage, this equation was solved for the unknown thickness t simultaneously for all temperatures, and the resulting thickness t was compared to the thickness of a ML of the adsorbates on Rh. If the kinetic energy of the emitted photoelectrons of the adsorbate and the metal differ by a sufficiently large amount, we cannot cancel out the photoelectron attenuation term of the equation, and need to calculate it separately for the adsorbate and the metal. The utilization of this equation is possible thanks to the small energy difference between the utilized Rh and adsorbate peaks. In order to estimate the error of this method we duplicated one of the measurements at a different incidence angle resulting in a different penetration depth, but without changing the actual coverage. This resulted in a deviation of the estimated coverage by a factor 0.015. Consequently, the relative coverages should be well representative.

The coverage of each fitted component, was on the other hand determined by utilizing a detailed spectrum of only the C 1*s* region. The estimated relative error for the coverage calculations is 17.2 %. The largest errors stem from the CH and CH_3 peaks

The total coverages of all carbon species during CO hydrogenation are given in Table S1.

Table S1. Coverages of carbon adsorbates during CO hydrogenation

Sample	Temperature (°C)	Tot Coverage (% of a ML)	Coverage CH - 284.0 eV (% of a ML)	Coverage CH_3 284.4 eV (% of a ML)	Coverage CH_3O 285.5 eV (% of a ML)	Coverage CO 286.0 eV (% of a ML)
Rh(111)	175	96.1	34.7	42.46	8.45	10.48
	200	91.6	38.9	32.17	9.69	10.85
	225	75.5	33.3	23.5	8.55	10.14
	250	28.3	10.8	4.11	4.33	8.98
	275	11.3	1.57	0.23	2.43	7.05
	300	12.0	0.75	0.56	2.01	8.68
	325	12.2	0.23	0.03	2.61	9.33
Rh(211)	175	80.5	8.99	53.6	9.75	8.12
	200	70.5	10.8	43.8	8.13	7.78
	225	55.9	9.3	32.8	5.92	7.93
	250	41.1	7.05	22.7	3.96	7.37
	275	32.0	4.6	9.7	2.46	6.28
	300	15.9	2.7	4.15	2.21	6.83
	325	11.3	1.44	2.31	1.54	5.99
	450	9.08	0.037	0.063	2.55	6.42

Hydrogen electron energy loss features

The photoelectrons emitted from the sample all have some probability to cause electronic excitations of the gas phase H_2 molecules. This excitation is visible as a satellite, with a maximum appearing at 12.8-13 eV away from every metal peak on the higher binding energy side and is mainly caused by excitation of the H_2 molecule to the 1Π ungerade state (sometimes just referred to as the 2p state, ΔE = ~ 12.5),[7] but excitations to higher states and into the vacuum also contribute. The multitude of excited states of the excited H_2 molecule give the peak a very broad and non-figurative shape. This hydrogen "ghost" peak has previously been observed and reported in XP spectra in ref.[8] Unfortunately for the case of studies on Rh catalysts, the Rh $3p_{1/2}$ component, located at 521.4 eV produce a hydrogen electron loss peak with a maximum at 534.3 eV which overlaps with the adsorbate region of oxygen and the majority of the intensity in the O 1*s* region originates from this electron energy loss (EEL). In order to extract oxygen coverages, we follow the steps illustrated in Figure S2. To begin with, in Figure S2 a) we see the entire Rh 3*p* and O 1*s* region. It comprises two Rh 3*p* peaks in blue, their corresponding hydrogen electron loss peaks as well as the gas phase peak. We modelled the Rh peaks and the gas phase peaks as Voigt shapes, the electron loss peaks as asymmetric pseudo Voigt shapes using the method by Schmidt et al[5] and were able to subtract a Tougaard background. After the subtraction we obtain the spectrum in Figure S2 b). We cropped the spectrum for the Rh $3p_{3/2}$ component and the $3p_{1/2}$ component, in the region highlighted by green and black. From the $3p_{1/2}$ component we subtract the dashed line which stems from the electron loss feature and from the $3p_{3/2}$ component we subtract the dotted line, which is the contribution from the 3/2 component. The cropped spectrum of the Rh $3p_{3/2}$ component is then scaled and shifted to the height and binding energy maximum of the 1/2 component. This results in Figure S1 c). The blue dotted data contains the overlapping adsorbate spectrum and the electron energy loss peak while the black line is a scaled and shifted peak of the 3/2 component and consequently does not have any adsorbates present. Subtracting these lines results in an isolated adsorbate spectrum.

The implementation of the Tougaard background modelling introduce a methodological error since areas of the electron loss peaks contribute to the modelled background, when they in reality do not contribute. Therefore, a quantification of the introduced error cannot easily be made. This is the reason why we prefer to not discuss the O 1*s* region in quantitative terms.

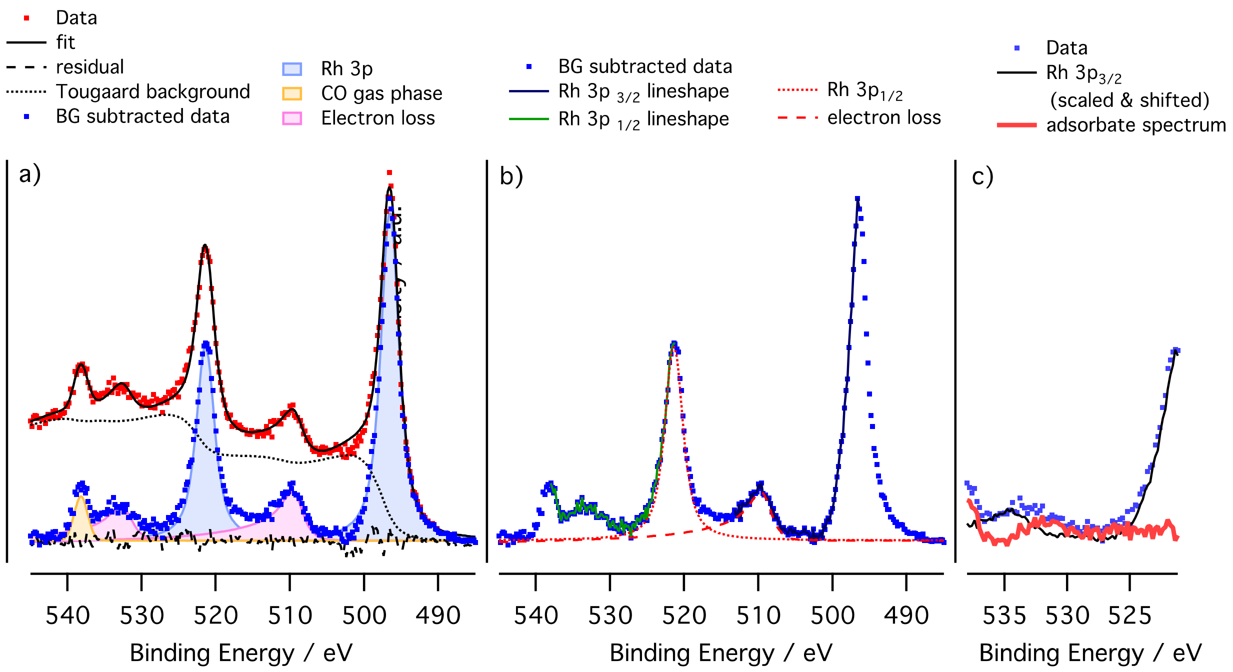

Figure S2. Illustration of the removal procedure which eliminates the contribution of the EEL peak from the O 1*s* spectra.

Supplementary Information

On binding energy corrections.

The reported binding energies in the main text are reported as measured after referencing to the Fermi edge, with no corrections to the presented data. Inherent to high energy (hard) x-ray experiments, some peaks may be shifted due to the recoil energy; i.e. the energy loss of the photoelectron due to the recoil from the emission process. This effect is less significant for conventional soft x-ray XPS performed with excitation energies of ~100 eV above the intended BE region. We have thus dedicated a section herein to estimate the magnitude of the said effect. The recoil energy E_R experienced by an atom in a solid upon photoionization is approximated using Equation S4.[9]

$$E_R \approx E_k m_e/M \qquad \text{Equation S4}$$

E_k is the kinetic energy of the electron and m_e/M is the mass ratio of the electron and the atom. For a photoelectron ejected from a C 1*s* orbital of a carbon adatom (B.E. 284) with the instrumentation described in the article we have a kinetic energy of 4312.5 eV and the mass ratio for a carbon atom and an electron is 4.56*10^{-5}. Since the binding energy is referenced to the Fermi edge, we also need to include the recoil energy of the valence band close to the Fermi level. The total shift due to recoil energy will thus be as shown in Equation S5:

$$\Delta E_R \approx E_{k(C1s)} m_e/M_C - E_{k(VB)} m_e/M_{Rh} \qquad \text{Equation S5}$$

Here the index VB denotes the valence band of the metal. The photoelectrons of the Fermi edge have a kinetic energy of 4596.5 eV and the mass ratio of the photoelectron to the Rh atom is 5.33*10^{-6}. The last piece of correction is to estimate the recoil shift of

our references as they seldom have accounted for recoil energy in their calculations either. The observed change in binding energy due to recoil energy compared to a soft x-ray reference system will be the difference between the systems as shown in Equation S6.

$$\Delta E_R \approx \left(E_{k(C1s,\,Exp)}{}^{m_e}/M_C - E_{k(VB,\,Exp)}{}^{m_e}/M_{Rh}\right) - \left(E_{k(C1s,\,Ref)}{}^{m_e}/M_C - E_{k(VB,\,Ref)}{}^{m_e}/M_{Rh}\right) \qquad \text{Equation S6}$$

where the indices Exp and Ref denote the experiment and reference respectively. The photon energy for measurements on the C 1*s* level used in reference[10] was 400 eV and will be used as an example. The kinetic energies assuming a 5 eV work function (a typical value as measured in our instrument) will be 106 eV for the C 1*s* photoelectron, and 395 eV for the valence band. Insertion of these values into equation S6 gives a shift of + 0.17 eV. The sign is significant; the recoil energy is a loss of photoelectron kinetic energy which means the binding energy is shifted in positive direction. As a carbon adatom is the smallest system considered in this paper the shift of all other species will be less than 0.17 eV. Furthermore, with the high-coverage-situation described in the main text, additional shifts due to coadsorption of several chemical species occur. The effect of coadsorption has for the case of CHx and CO on Rh(111) been investigated by DFT, which showed that the binding energies of CHx species can shift in either positive or negative directions up to 0.4 eV.[11]

Justification for peak assignments

C 1*s* peak assignments

The assignments of the peaks are made on a basis of experimental references from literature, calculated shifts from DFT and/or deductive reasoning. To begin with, we will discuss the C 1*s* peak assignments. In the literature there are a few different assignments to the CH_x species with varying credibility. An often cited paper by Veselli and coworkers is one of few synchrotron-based reference studies which, amongst other, identifies two peaks around 283.3 eV and 283.7 eV. The 283.3 eV peak is assigned to atomic C, while the second one could be either CH or an inequivalent variant of atomic C.[10] Other references with far more noisy spectra originate from Iodine substituted methane in experiments by Solymosy and coworkers[12,13], which tend to note a temperature and coverage dependence of the peak position. At the examined temperatures and coverages most relevant to our study they identify CH_3 at 284.6 eV and, the CH_2 specie at 283.7 eV. It is unanimously agreed upon that more hydrogen saturated hydrocarbons result in higher binding energy, as this has been confirmed by DFT calculations which are notoriously bad for calculating exact spectroscopic energies, but work well for examining trends. We have identified the two peaks clearly identifiable at 284.0 eV and 284.4 eV. From DFT calculations on the reaction pathway for CH_4 dissociation we know that the two reaction sinks are the CH_3 and CH species, which thus are the two most likely contributors. As was mentioned in the discussion segment of the main text, we know that multicarbon molecules also contribute to the C 1*s* spectra at the lower temperatures since the total C coverage exceeds 75 % of a ML, which is unlikely unless multicarbon species are present.

It is also worth mentioning that sp2-hybridized carbon, such as graphite, (poly-) aromatic compounds and triple-bond containing intermediates such as ethyildyne. There is a range between 284.7 eV and 285.3 eV in Figure 1 of the main text which clearly contains spectrum intensity, but is not highlighted with any identifier. At first glance it might look reasonable to assign this region to graphitic or aromatic carbon, as the binding energies would match, but we do not believe that there is a major contribution from these compounds. The reason is that the shape of the intensity in this region does not align with any aromatic or graphitic type of carbon. These molecules have several symmetric and spectroscopically equivalent carbons and would have resulted in a sharp and narrow peak, and this is the opposite of what we observe as the region does not contain any peak or shoulder at all. The region looks more like a tailing of the CH_x peaks, which is a more plausible explanation considering that it is common for chemisorbed hydrocarbons on metal surfaces to produce shake-up features in XPS.

CO, on the contrary, is well-referenced in the literature and it has been agreed upon that there is an on-top peak with a maximum at 286 eV and a hollow peak at 285.3 eV. There is to the authors knowledge no reference spectrum for the methoxy species on Rh, as Rh is seldom used as a catalyst for methanol synthesis, but on other surfaces (i.e. copper) we have reliable references, which point toward a binding energy of 285.3 eV, and a shifting towards higher binding energy at higher coverages.[14] Normally, the Cu and Rh surfaces have fundamentally different electronic d-band filling which drastically change the binding energy of the adsorbates bound to them, but in the case of chemisorbed methoxy radicals this is less of an issue since the methoxy species are bound to the metal through the oxygen atom, rather than the carbon. We found a peak at 285.5 eV, which clearly has too high binding energy to be CO in the hollow site, moreover, the CO in hollow peak tends to emerge only at coverages above 33% of a ML, but the shoulder is still there even at coverages below 30%. We must thus assume another species and the methoxy radical is the next stable intermediate with a close-lying binding energy. Other potential species to fill this binding energy range would include CHO, COH, CHOH, but time-resolved pump-probe experiments have found that these intermediates are generally very short-lived and are consequently not expected to contribute significantly.[15]

Table S2. Justification of XP peaks, CO hydrogenation

Peak Binding Energy (eV)	Justification for peak	Main Hypothesis	Other adsorbates that contribute to a lower degree
284.0	Peak maximum at 200°C on the (111) surface	CH,	C and CH_2
284.4	Peak maximum at 200°C on the (211) surface	CH_3, C-C bonded carbon	sp2-hybridized carbon
285.5	Peak maximum of left component below 250°C on Rh(111)	CH_3O	COH, CHO, CHOH
28.6.0	Peak maximum above 300°C at both surfaces	CO on-top	-

On beam induced effects

The CO and CO_2 hydrogenation reactions are inherently susceptible to beam induced effects, sometimes referred to as beam damage. Three mechanisms for beam damage have been considered for this experiment. Firstly, we have direct heating of the sample by the x-ray beam. This has been estimated to only account for a very small degree of heating, and is readily offset by the cooling of the gas stream. Secondly, we have direct excitations of the molecules by the x-ray beam. Even though a narrow focus and high flux of the beam was used we note that a double excitation of the adsorbate is unlikely. The third mechanism is excitation of gas and adsorbate molecules due to photoelectrons from the metal substrate. The excited gas phase molecules have substantially lower barriers for dissociation, and are thus more reactive. This can show in the adsorbate spectrum as a flux dependence of the line shapes and even as the addition of extra peaks with increased flux. The combination of hard x-rays, the high pressure and high flux makes this mechanism the most severe form of beam damage. We know from experience that the C 1*s* region is most sensitive to beam damage, and we have attenuated the beam until the C 1*s* spectrum stopped changing. With a base flux of ~13^{10} photons/second and 10x10 μm we noted that an attenuation factor of 5.5 was necessary to offset beam induced effects for the CO hydrogenation systems and a factor of 11 was necessary for the CO_2 hydrogenation systems.

Online mass spectrometer data

Mass spectrometer (MS) data for selected masses acquired during CO hydrogenation over the Rh(211) surface are shown in Figure S3, and for CO_2 hydrogenation on the Rh(211) surface the corresponding data is available in figure S4. This is thus acquired during the acquisition of the spectra in Figure 1 b) and Figure 3 b) of the main text respectively. The m/z ratios 2, 28 and 44 correspond to hydrogen, CO and CO_2 respectively, while m/z= 15, 18, 30 and 31 all correspond to potential products of the CO or CO_2 hydrogenation reaction. m/z = 15 is the methyl radical, which is a prevalent part of the MS pattern for methane. We can unfortunately not use the strongest peak of the CH_4 mass spectrum (m/z = 16) as it overlaps with the oxygen radical which is a prevalent feature of the CO pattern. m/z = 18 is the molecular weight of water, and also the most prevalent peak in the water mass spectrum. m/z = 30 is the mass for ethane (C_2H_6), but influences from the natural occurrences of ^{14}CO, $^{13}C^{17}O$ and $C^{18}O$ may contribute to this peak. Once again, the strongest peak in the ethane pattern is m/z = 28, which cannot be used as it overlaps with the reactant CO. Lastly, m/z = 31 correspond to the methoxy radical (CH_3O) indicative of methanol production. We cannot use the m/z = 32 signal due to overlap from a tiny contamination in the gas stream from atmospheric oxygen.

It is clear from Figure S3 that most product signals do not change drastically with sample temperature. The largest exception is the water signal at m/z = 18. It should be noted, however, that the water signal is affected by the accumulation onto the chamber walls during transport from the sample region to the mass spectrometer and thus tends to increase or decrease steadily over time as the reaction is running. Other than the water signal there is a small increase of the methoxy signal at m/z = 31.

In Figure S4, on the other hand, there is no significant change of the MS signal of any of the products, except for the water signal which – as discussed previously – does not necessarily relate to the actual activity of the reaction.

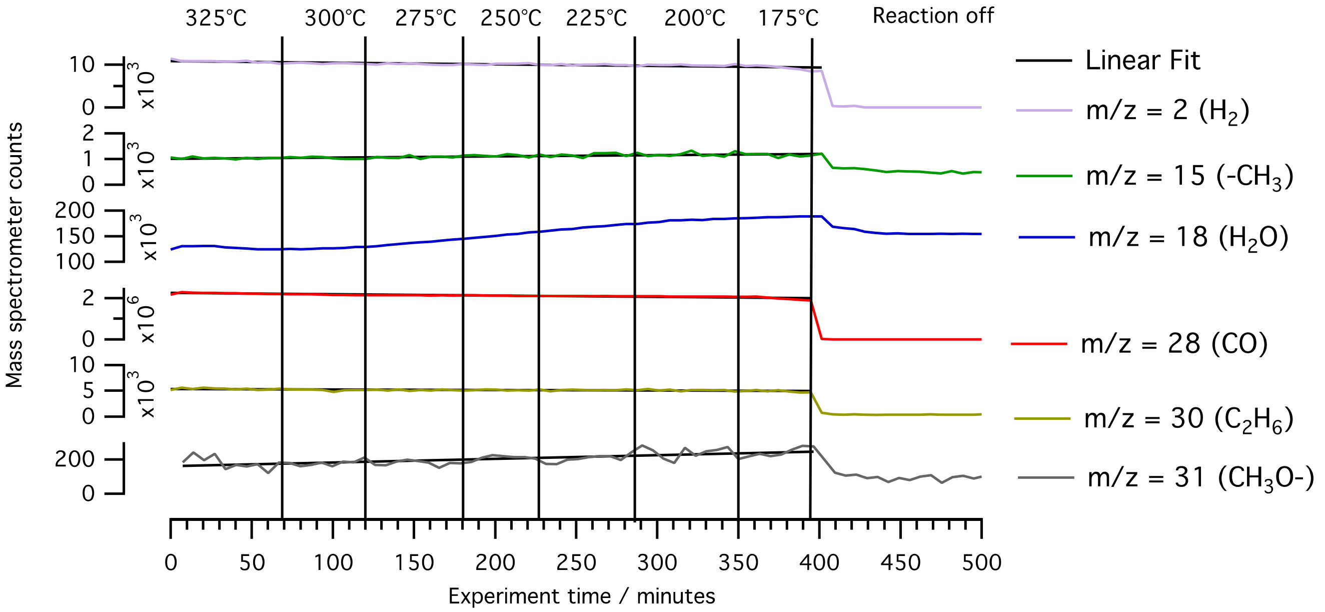

Figure S3. Development of selected mass signals with experiment time during CO hydrogenation over the Rh(211) surface.

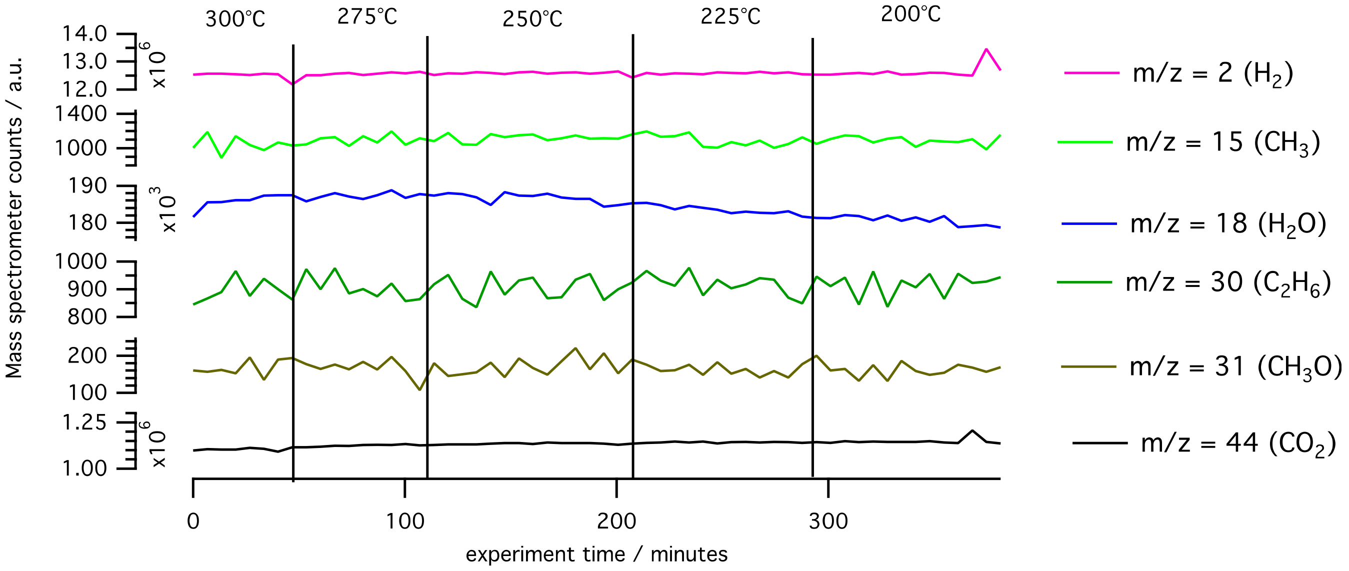

Figure S4. Development of selected mass signals with experiment time during CO_2 hydrogenation over the Rh(211) surface.

The large flows required in order to maintain the pressure at the sample region will create inconveniences for the detection of reaction products. We need to consider the minimum turnover frequency (TOF) required in order to detect products. This quantity can be estimated from known experimental parameters. The TOF is defined as the number of products (n_p) per active site and second as written in equation S7.

$$TOF = \frac{n_p}{n_{sites} * t_d} \qquad \text{Equation S7}$$

where n_{sites} is the number of active sites and t_d is the dwell time of the molecules in the uptake volume. From the calibrated pressure and flow ratings we estimate that the 0.053 mm3 uptake volume contains circa $3.67*10^{13}$ CO molecules at any given point

and the dwell time is circa $1.29*10^{-4}$ seconds. We assume that on the (211) surface the limiting active sites for the reaction are the fourfold sites required for CO dissociation, which occur once per surface unit cell. This means that the (211) surface contains $9.97*10^{12}$ sites within the sampling region.

In order for a change in the MS signal to be detected it has to be changed by 3x the noise level, the noise level being defined as 2x the standard deviation of the signal. Out of the reactions examined in this paper the most active is supposedly CO hydrogenation on the (211) surface. From the MS data we see that the average CO signal at m/z = 28 when the reaction was turned on was $2.13*10^6$ a.u. Investigating the signal for the potential products, we see that the noise level of m/z =15 was 162 a.u. and in order for the change in this signal to reach the limit of detection it has to increase by 162*3 = 486 a.u. We call the quotient between the lowest detectable amount of product signal and the reactant signal the "lowest detectable fraction" (LDF) and this quantity evaluates to $486/2.13*10^6 = 2.29*10^{-4}$. We work under the assumptions (1) that the products and the CO molecules are present to the same degree in the sampling volume and in the MS detector volume and (2) that the signal is proportional to the number of molecules, with the same proportionality for both CO and CH_4. With these assumptions we can estimate the number of turnovers needed in order for the reaction to be detected as the LDF multiplied by the number of CO molecules in the sampling volume, i.e. $2.29*10^{-4} * 3.67*10^{13} = 7.49*10^{10}$. This we can insert into equation S7 which yields equation S8

$$TOF = \ = \frac{8.39\ \times 10^9\, TOs}{9.97 \times 10^{12}\, sites *\ 1.29 * 10^{-4} s} = 6.5\, site^{-1} s^{-1} \qquad \text{Equation S8}$$

The resulting value of 6.5 turnovers per site and second is higher than the expected turnover frequency from our system, considering that previous measurements on the TOFs for Rh single crystals and foils typically amount to 0.1 - 1.5 per second and site, even when measured at considerably higher pressures than in this study.[16] Thus, while the change in signal is not expected to reach above the limit of detection, the slope of the signal may still be statistically significant. We have thus fitted the spectra with a linear model in order to clarify any increases or decreases in the signal. The slope and the corresponding standard deviation are shown in Table S3. In the case for m/z = 31 we noted an increase in the signal at lower temperatures, which in the table is shown as a positive slope. The increase of m/z signal coincides with the appearance of the methoxy species in the O 1*s* and C 1*s* spectra. While weak, the slope is statistically significant and opposes the slope of the reactant gases, indicating a possibility that some methanol is being produced. The same is true for the m/z = 15 signal, which corresponds to the methyl radical and is part of the mass spectra of all alkanes.

It is interesting to note that the lower temperatures are seemingly more active than the higher temperatures, which would indicate that the limiting factor for turnovers are not related to the energy barriers of the reaction but rather to the residence time of the reactants on the surface. We note that this might be different at higher pressures.

Table S3. Slope of fitted line with MS signal

m/z ratio	Main constituent	Slope (a.u. / min)	Standard deviation of slope
2	H_2	-3.64	0.31
15	CH_3-	0.46	0.06
28	CO	-604	35.8
30	C_2H_6	-0.95	0.17
31	CH_3O-	0.21	0.03

All O 1*s* spectra

Due to space restrictions and focus on the relevant results we have omitted the O 1*s* spectra acquired during the measurement from the main text. In this section we discuss the O 1*s* spectra and how they support the findings of the main text. The adsorbate signal is very broad compared to the C 1s region, and the overlap with the EEL region makes the interpretation harder. Figure S5 displays all spectra of the O 1*s* region during CO hydrogenation on Rh(111). Subplot a) plots the spectra as acquired, including the overlapping

EEL peak. For comparison, each of the spectra are compared to the 325°C spectrum. Subplot b) shows the difference spectrum between 325°C and each other temperature, with a Savitzky-Golay smoothening algorithm applied. This clarifies at which binding energies the spectra differ, and it removes the EEL contribution, but this treatment also subtracts the parts of the spectra that do not change with temperature. For this reason, we have also plotted an EEL subtracted spectrum in subplot c), using the method discussed in the "hydrogen electron energy loss" section. We make the following observations: in the difference spectra as well as in the raw spectra we note enhanced intensity around 530.7 eV for the lower temperatures. This corresponds well to the binding energy of the methoxy species, and the decreasing trend of the peak agrees with the similar temperature behavior of the methoxy peak in the C 1*s* spectrum. Around 532 eV where one would expect CO to appear, there is little-to-no difference between the spectra at different temperatures. But, as was seen in the C 1*s* spectra, this is expected, since the CO coverage was also not changing significantly in the C 1*s* spectra either. In the EEL-subtracted spectrum we note that the maximum intensity is located around 532 eV, indicating that the majority of the oxygen coverage stems from CO.

Similarly, in Figure S6 we have made the same treatment for the O 1*s* region on Rh(211). As was the case for the (111) surface orientation we note a that the most significant contribution to the O 1*s* spectrum in Figure S6c) once again stems from CO as seen by a peak maximum at 532 eV. This contribution from CO is lower than on the (111) surface, which is also consistent with the C 1*s* spectra. What is not consistent, on the other hand is the coverage of methoxy on the Rh(211) surface, as we cannot see the same trend in the 530.7 region as we did for the (111) surface. The very low coverage in general is likely the cause of this discrepancy. Using the Rh 3p ½ component as a standard, we estimate the total O 1*s* coverage on the Rh(111) to amount to 25% and the total coverage on the Rh(211) surface to around 15%.

In Figure S7, we have displayed the O 1*s* region during CO_2 hydrogenation. We note, similar to the C 1s region, a higher coverage on the (211) surface, most evident in Figure S7 c). We also note that the 530 (O_{ads}), 530.7 (CH_3O eV) and 532 (CO) regions differ in coverage between the CO and CO_2 hydrogenation reaction as seen in Figure S7 d).

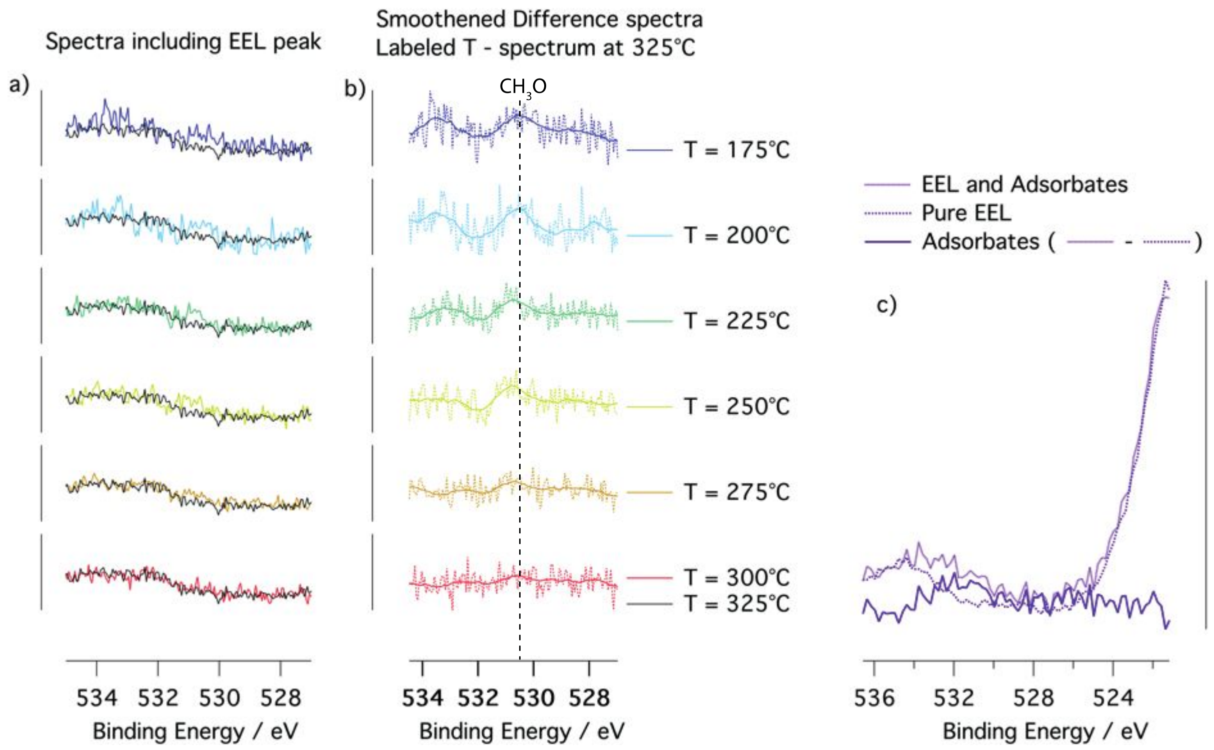

Figure S5. XP spectra of the O 1*s* region during ongoing CO hydrogenation on the Rh(111) surface. The pressure was 150 mbar, the $CO:H_2$ ratio was 1:2, and the photon energy was 4.6 keV.

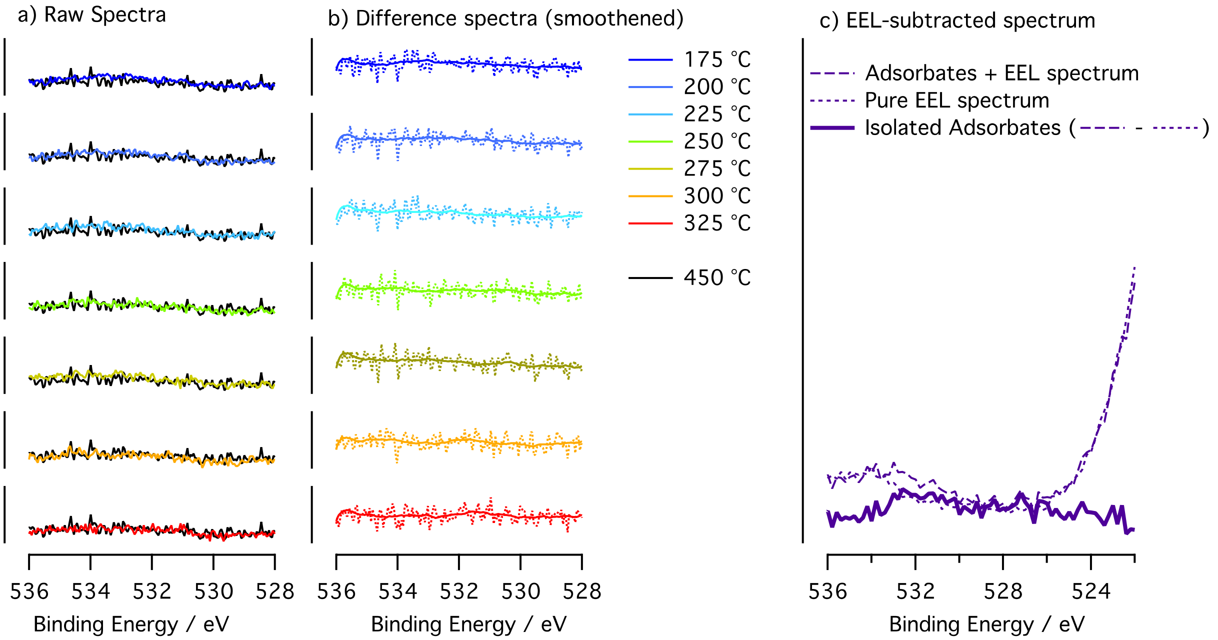

Figure S6. XP spectra of the O 1*s* region during ongoing CO hydrogenation on the Rh(211) surface. The pressure was 150 mbar, the CO:H_2 ratio was 1:2, and the photon energy was 4.6 keV.

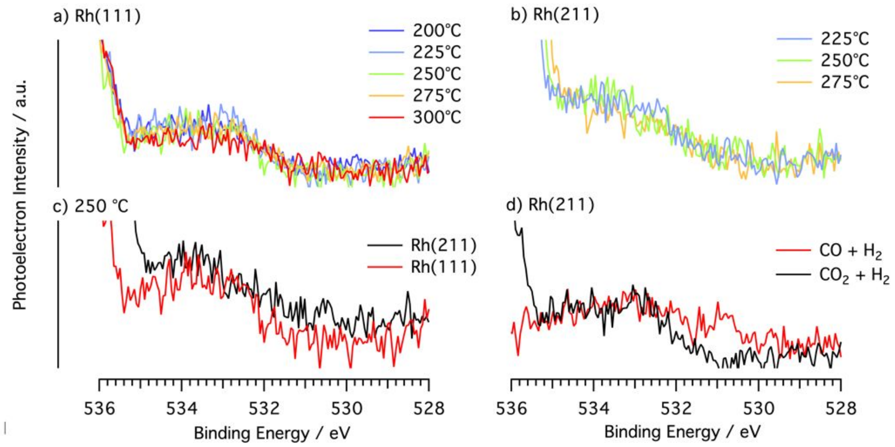

Figure S7. XP spectra of the O 1*s* region during ongoing CO_2 hydrogenation on a) the Rh(111) surface, and b) the Rh(211) surface. Subplot c) illustrates the difference between the two surfaces for the measurements at 250°C, and subplot d) contains a comparison of the XP spectra recorded during the CO hydrogenation reaction and the CO_2 hydrogenation reaction at 250°C on the Rh(211) surface. The total pressure was 150 mbar, the CO_2:H_2 ratio was 1:2, and the photon energy was 4.6 keV.

Spectra of the Rh 3*d* region

Spectra of the Rh 3*d* region are shown in Figure S8. As a function of temperature, minute changes to the Rh 3*d* region were observed, mainly at the lower binding energy side of the 5/2 component. At all examined conditions, the Rh spectrum remained metallic. For all systems, the intensity around 306.6 eV increases with temperature. Since the coverage is temperature-dependent, we conclude that these intensity changes occur due to a surface state which at higher temperatures becomes accessible due to a lower coverage of hydrogen, carbon and oxygen compounds and this is consistent with other experiments where the surface-core-level

shifts have been resolved in vacuum.[17] The shoulder barely visible at 315.8 eV in the CO-containing spectra occurs due to electron losses from excitations of the CO molecule.

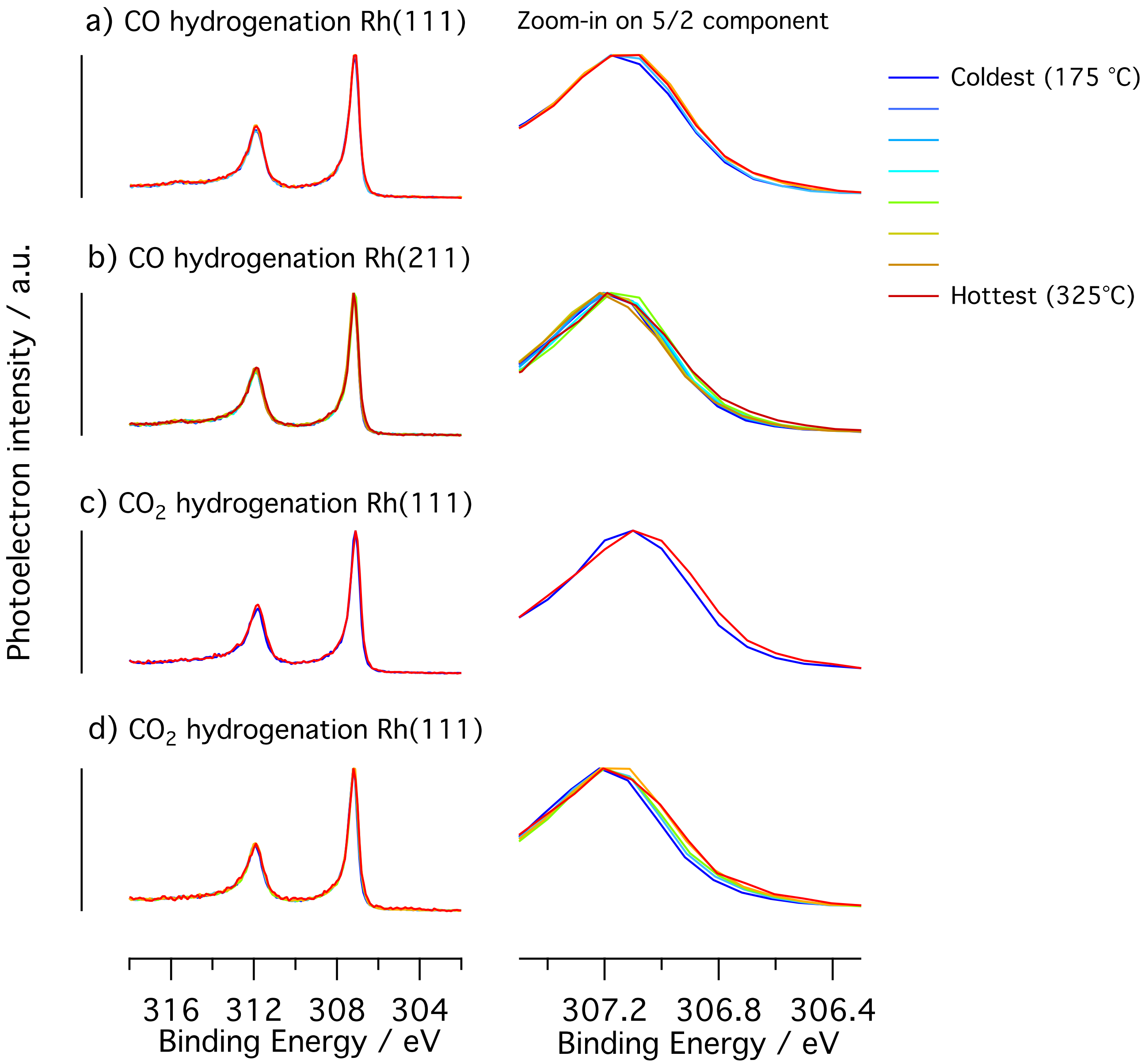

Figure S8. XP spectra of the Rh 3*d* region during a) CO hydrogenation on Rh(111), b) CO hydrogenation on Rh(211), c) CO2 hydrogenation on Rh(111) and d) CO2 hydrogenation on Rh(211). The right hand side shows a zoom-in of the 5/2 component.

Fits of the C 1*s* spectra during CO hydrogenation.

All fits of the CO hydrogenation peaks used for the graph in Figure 2 of the main text are shown in Figure S9 for Rh(111) and in figure S10 for Rh(211). The raw data (without the Shirley background subtraction) is displayed in Figure 1 of the main text.

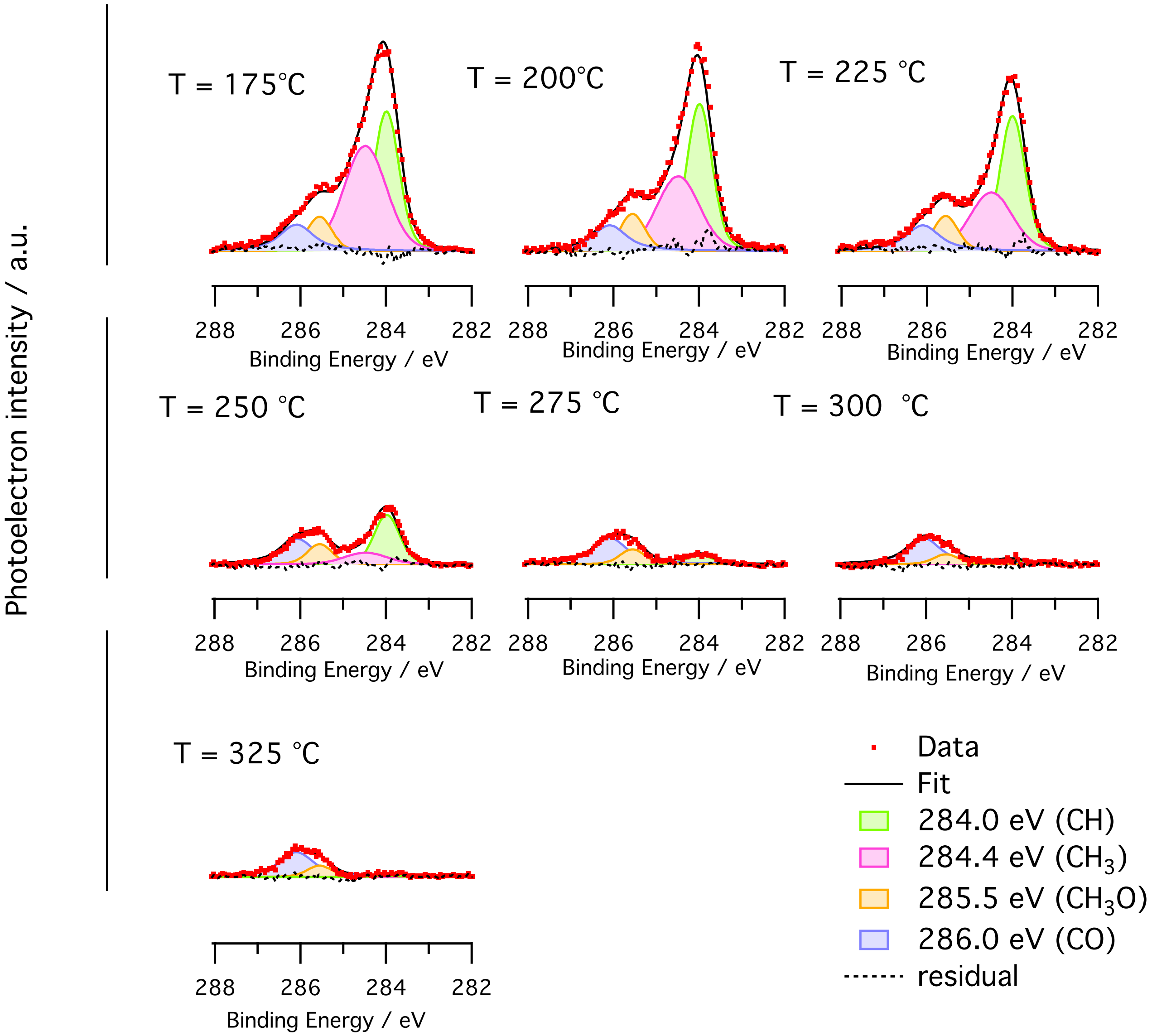

Figure S9. Fitted XP spectra of C 1*s* region during CO hydrogenation on Rh(111), which was used as the basis for the coverage calculations of the individual components as shown in Figure 2a) of the main text as well as Table S1 in this Supplementary Material.

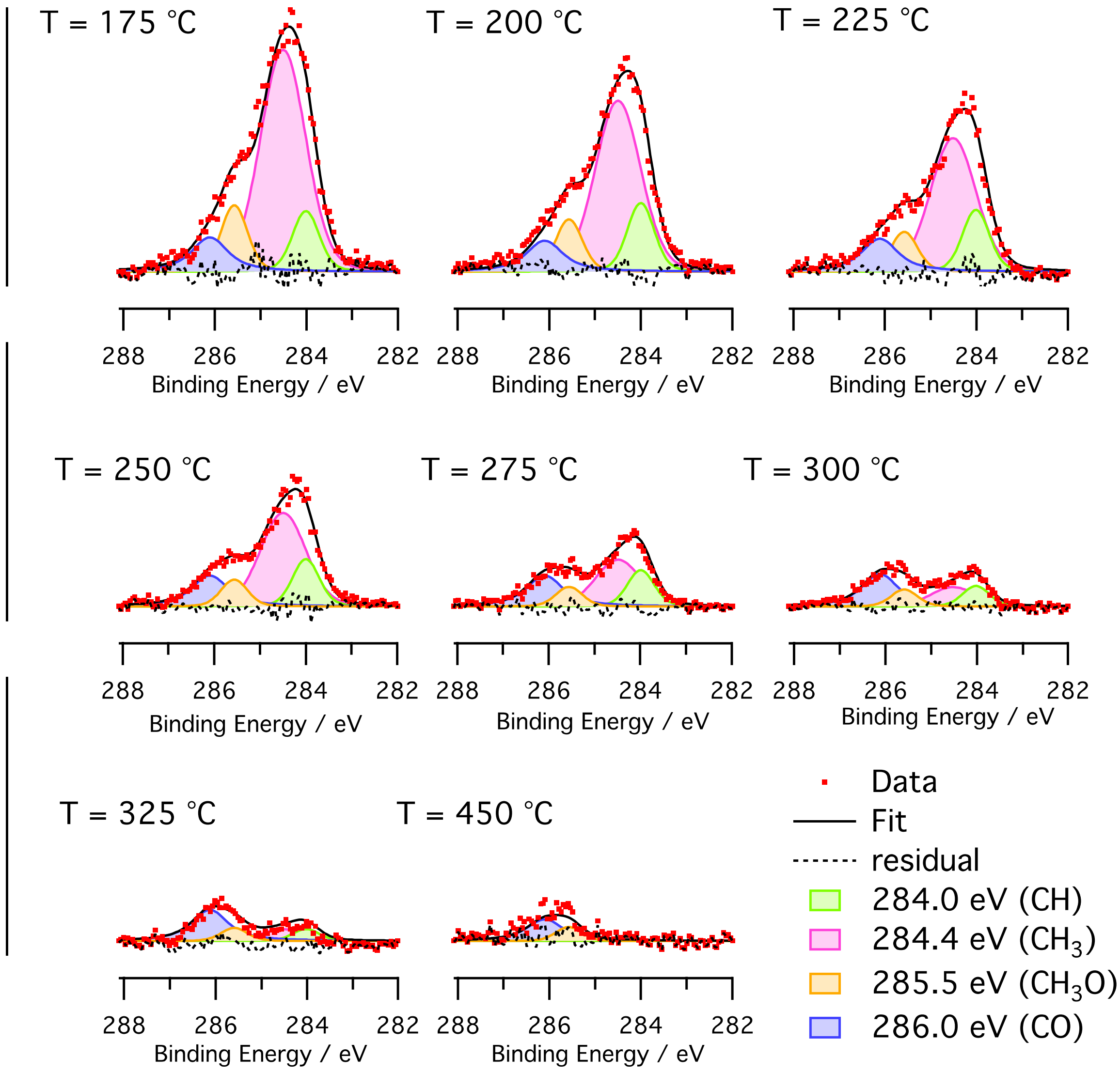

Figure S10. Fitted XP spectra of C 1*s* region during CO hydrogenation on Rh(211), which was used as the basis for the coverage calculations of the individual components as shown in Figure 2b) of the main text as well as Table S1 in this Supplementary Material.

REFERENCES

(1) Amann, P.; Degerman, D.; Lee, M. T.; Alexander, J. D.; Shipilin, M.; Wang, H. Y.; Cavalca, F.; Weston, M.; Gladh, J.; Blom, M.; Björkhage, M.; Löfgren, P.; Schlueter, C.; Loemker, P.; Ederer, K.; Drube, W.; Noei, H.; Zehetner, J.; Wentzel, H.; Åhlund, J.; Nilsson, A. A High-Pressure x-Ray Photoelectron Spectroscopy Instrument for Studies of Industrially Relevant Catalytic Reactions at Pressures of Several Bars. *Rev. Sci. Instrum.* **2019**, *90* (10). https://doi.org/10.1063/1.5109321.

(2) Goodwin, C.; Alexander, J.; Weston, M.; Degerman, D.; Shipilin, M.; Loemker, P.; Amann, P. EXPRESS: A Novel Method to Maintain the Sample Position and Pressure in Differentially Pumped Systems Below the Resolution Limit of Optical Microscopy Techniques. *Appl. Spectrosc.* **2020**, 000370282094279. https://doi.org/10.1177/0003702820942798.

(3) Parratt, L. G. Surface Studies of Solids by Total Reflection of X-Rays. *Phys. Rev.* **1954**, *95* (2), 359–369. https://doi.org/10.1103/PhysRev.95.359.

(4) Shipilin, M. specqp https://github.com/Shipilin/specqp.

(5) Schmid, M.; Steinrück, H. P.; Gottfried, J. M. A New Asymmetric Pseudo-Voigt Function for More Efficient Fitting of XPS Lines. *Surf. Interface Anal.* **2014**, *46* (8), 505–511. https://doi.org/10.1002/sia.5521.

(6) Goodwin, C. M.; Shipilin, M.; Albertin, S.; Hejral, U.; Lömker, P.; Wang, H. Y.; Blomberg, S.; Degerman, D.; Schlueter, C.; Nilsson, A.; Lundgren, E.; Amann, P. The Structure of the Active Pd State during Catalytic Carbon Monoxide Oxidization. *J. Phys. Chem. Lett.* **2021**, *12* (18), 4461–4465. https://doi.org/10.1021/acs.jpclett.1c00620.

(7) Huber, K.-P.; Hertzberg, G. *Constants of Diatomic Molecules*; Huber, K.-P., Hertzberg, G., Eds.; Van Nostrand Reinhold Publishing: New York,

1979; Vol. 4. https://doi.org/10.1016/0022-2860(80)80150-5.
(8) Teschner, D.; Pestryakov, A.; Kleimenov, E.; Hävecker, M.; Bluhm, H.; Sauer, H.; Knop-Gericke, A.; Schlögl, R. High-Pressure X-Ray Photoelectron Spectroscopy of Palladium Model Hydrogenation Catalysts.: Part 1: Effect of Gas Ambient and Temperature. *J. Catal.* **2005**, *230* (1), 186–194. https://doi.org/10.1016/j.jcat.2004.11.036.
(9) Takata, Y.; Kayanuma, Y.; Yabashi, M.; Tamasaku, K.; Nishino, Y.; Miwa, D.; Harada, Y.; Horiba, K.; Shin, S.; Tanaka, S.; Ikenaga, E.; Kobayashi, K.; Senba, Y.; Ohashi, H.; Ishikawa, T. Recoil Effects of Photoelectrons in a Solid. *Phys. Rev. B - Condens. Matter Mater. Phys.* **2007**, *75* (23), 1–4. https://doi.org/10.1103/PhysRevB.75.233404.
(10) Vesselli, E.; Baraldi, A.; Comelli, G.; Lizzit, S.; Rosei, R. Ethanol Decomposition: C-C Cleavage Selectivity on Rh(111). *ChemPhysChem* **2004**, *5* (8), 1133–1140. https://doi.org/10.1002/cphc.200400043.
(11) Yang, M. M.; Bao, X. H.; Li, W. X. Density Functional Theory Study of CHx (X=1-3) Adsorption on Clean and CO Precovered Rh(111) Surfaces. *J. Chem. Phys.* **2007**, *127* (2). https://doi.org/10.1063/1.2751155.
(12) Solymosi, F. Thermal Stability and Reactions of CH2, CH3and C2H5species on the Metal Surfaces. *Catal. Today* **1996**, *28* (3), 193–203. https://doi.org/10.1016/0920-5861(95)00242-1.
(13) Klivényi, G.; Solymosi, F. Generation of CH2 Species: Thermal and Photo-Induced Dissociation of CH2I2 on Rh(111) Surface. *Surf. Sci.* **1995**, *342* (1–3), 168–184. https://doi.org/10.1016/0039-6028(95)00767-9.
(14) Orozco, I.; Huang, E.; Mahapatra, M.; Kang, J.; Shi, R.; Nemšák, S.; Tong, X.; Senanayake, S. D.; Liu, P.; Rodríguez, J. A. Understanding Methanol Synthesis on Inverse ZnO/CuOx/Cu Catalysts: Stability of CH3O Species and Dynamic Nature of the Surface. *J. Phys. Chem. C* **2021**, *125* (12), 6673–6683. https://doi.org/10.1021/acs.jpcc.1c00392.
(15) LaRue, J.; Krejčí, O.; Yu, L.; Beye, M.; Ng, M. L.; Öberg, H.; Xin, H.; Mercurio, G.; Moeller, S.; Turner, J. J.; Nordlund, D.; Coffee, R.; Minitti, M. P.; Wurth, W.; Pettersson, L. G. M.; Öström, H.; Nilsson, A.; Abild-Pedersen, F.; Ogasawara, H. Real-Time Elucidation of Catalytic Pathways in CO Hydrogenation on Ru. *J. Phys. Chem. Lett.* **2017**, *8* (16), 3820–3825. https://doi.org/10.1021/acs.jpclett.7b01549.
(16) Castner, D. G.; Blackadar, R. L.; Somorjai, G. A. CO Hydrogenation over Clean and Oxidized Rhodium Foil and Single Crystal Catalysts. Correlations of Catalyst Activity, Selectivity, and Surface Composition. *J. Catal.* **1980**, *66* (2), 257–266. https://doi.org/10.1016/0021-9517(80)90030-5.
(17) Kim, J.; Ha, H.; Doh, W. H.; Ueda, K.; Mase, K.; Kondoh, H.; Mun, B. S.; Kim, H. Y.; Park, J. Y. How Rh Surface Breaks CO 2 Molecules under Ambient Pressure. *Nat. Commun.* **2020**, *11*, 5649. https://doi.org/https://doi.org/10.1038/s41467-020-19398-1.
